# Methylarginine Levels in Chronic Inflammatory Skin Diseases—The Role of L-Arginine/Nitric Oxide Pathway

**DOI:** 10.3390/jcm14227934

**Published:** 2025-11-08

**Authors:** Clara Matei, Mircea Tampa, Madalina Irina Mitran, Cristina Iulia Mitran, Ilinca Nicolae, Corina Daniela Ene, Andrei Marin, Ecaterina Rinja, Adrian Dumitru, Constantin Caruntu, Carolina Constantin, Monica Neagu, Simona Roxana Georgescu

**Affiliations:** 1 Department of Dermatology, ‘Carol Davila’ University of Medicine and Pharmacy, 020021 Bucharest, Romania; clara.matei@umfcd.ro (C.M.); dermatology.mt@gmail.com (M.T.); simona.georgescu@umfcd.ro (S.R.G.); 2 Department of Dermatology, ‘Victor Babes’ Clinical Hospital for Infectious Diseases, 030303 Bucharest, Romania; drnicolaei@yahoo.ro; 3 Department of Microbiology, ‘Carol Davila’ University of Medicine and Pharmacy, 020021 Bucharest, Romania; 4 Departments of Nephrology, ‘Carol Davila’ University of Medicine and Pharmacy, 020021 Bucharest, Romania; corina.ene@umfcd.ro; 5 Department of Nephrology, ‘Carol Davila’ Nephrology Hospital, 010731 Bucharest, Romania; 6 Plastic Surgery Department, St. John’s Hospital, Carol Davila University, 042122 Bucharest, Romania; andrei.marin@umfcd.ro; 7 Gastroenterology Department, Clinical Emergency Hospital of Bucharest, 014461 Bucharest, Romania; ecaterina.rinja@gmail.com; 8 Department of Pathology, ‘Carol Davila’ University of Medicine and Pharmacy, 020021 Bucharest, Romania; vasile.dumitru@umfcd.ro; 9 Department of Pathology, University Emergency Hospital, 050098 Bucharest, Romania; 10 Department of Physiology, ‘Carol Davila’ University of Medicine and Pharmacy, 050474 Bucharest, Romania; costin.caruntu@gmail.com; 11 Department of Dermatology, “Prof. N.C. Paulescu” National Institute of Diabetes, Nutrition and Metabolic Diseases, 011233 Bucharest, Romania; 12 Faculty of Biology, University of Bucharest, Splaiul Independentei 91–95, 050095 Bucharest, Romania; caroconstantin@gmail.com (C.C.); neagu.monica@gmail.com (M.N.); 13 Immunology Department, “Victor Babes” National Institute of Pathology, 050096 Bucharest, Romania

**Keywords:** L-arginine, nitric oxide, methylarginines, skin diseases

## Abstract

In recent years, the L-arginine/nitric oxide (NO) pathway has garnered increasing attention across a range of pathological conditions, including skin diseases. NO is an important modulator of skin homeostasis, being actively involved in numerous processes such as vasodilation, keratinocyte proliferation, melanogenesis and cell signaling. Under inflammatory conditions, post-translational changes in L-arginine take place, resulting in the synthesis of methylarginines including monomethylarginine (MMA), asymmetric dimethylarginine (ADMA), and symmetric dimethylarginine (SDMA). Once ADMA and MMA are generated, they compete with L-arginine to bind to the active site of NO synthase, which reduces the production of NO. Additionally, SDMA inhibits the transport of L-arginine, leading to a lower concentration of this amino acid within cells. Consequently, by impacting both the availability of L-arginine and the production of NO, conditions favoring oxidative stress and endothelial dysfunction are created. Dysregulation of L-arginine/NO pathway is closely related to inflammation and oxidative stress, two events that play a cardinal role in the pathogenesis of chronic inflammatory skin diseases. We conducted a narrative review that synthesizes current evidence on methylarginine levels in patients with chronic inflammatory skin diseases. Our aim was to enhance our knowledge about the role of these compounds in pathogenesis and provide new insights into the mechanisms underlying these conditions that can be the basis for novel diagnostic biomarkers and therapies.

## 1. Introduction

L-arginine serves as a substrate for several enzymes, the most important of which is NO synthase (NOS) [1]. Three isoforms of NOS have been described (two constitutive (cNOS)—endothelial and neuronal—and one inducible (iNOS)) so far. These enzymes participate in the generation of nitric oxide (NO) and L-citrulline. cNOS synthesises NO through a calcium-dependent pathway in small amounts that are necessary for physiological processes. iNOS is expressed by cells that modulate the inflammatory response and, under the action of cytokines through a calcium-independent pathway, promotes the release of increased amounts of NO. Additionally, L-arginine metabolism occurs under the action of arginases, resulting in urea and L-ornithine [2,3]. NO is a short-lived molecule whose role has been extensively characterised in cardiovascular diseases, influencing blood pressure and vascular resistance. In fact, NO is a gaseous molecule that ensures homeostasis in numerous organs, including the heart, brain, kidneys, skin, etc. It is a versatile compound that modulates processes such as inflammation, vasodilation, and cell proliferation [4,5]. NO is synthesized by numerous cells in the human body, including endothelial cells, immune cells, liver cells, keratinocytes, and fibroblasts [6]. NO plays a key role in maintaining homeostasis in the skin, mediating various events such as skin cell proliferation (keratinocytes, fibroblasts) or the inflammatory/immune response by promoting T cell proliferation. Significant advancements have been made in measuring NO levels in recent years. The physiological concentration of NO typically ranges from 100 picomolars (pM) to 5 nanomolars (nM). In skin inflammatory processes, NO can have a dual role; it has been found to act both as a pro-inflammatory mediator and as an inhibitor of inflammatory cells [7]. In the skin, at low levels, NO serves as a signaling molecule that contributes to the regulation and maintenance of physiological balance, playing roles in processes such as blood vessel dilation, melanin production, and defense against environmental stressors. At elevated levels, NO can have detrimental effects on cellular components and is associated with immune-mediated inflammatory skin disorders, such as psoriasis, lupus erythematosus, and allergic skin reactions [4,5,8,9,10].

Arginine can undergo three forms of methylation resulting in three main methylarginines: monomethylarginine (MMA), asymmetric dimethylarginine (ADMA), and symmetric dimethylarginine (SDMA). Protein arginine methyltransferases (PRMTs) play the main role in the synthesis of these compounds, with certain types producing a single methylation resulting in MMA, and other types producing a second methylation resulting in ADMA and SDMA [11]. PRMT1 is involved in mediating inflammation and during inflammation, PRMT1 negatively affects the nuclear factor kappa B (NF-κB) pathway and this phenomenon stops the activation of NF-κB target genes promoters. The involvement of NF-κB in inflammation and in the immune responses is unquestionable, being NF-κB’s most important role. PRMT1 suppresses class II trans-activator (CIITA)-mediated MHC-II transactivation. When Pattern recognition receptors (PRR) are stimulated by γ interferon-gamma (IFN-γ) asymmetric dimethylation of CIITA occurs. CIITA becomes susceptible to degradation and it does not translocate to the nucleus and hence increase MHC-II genes expression. The inhibition of factors that deregulate cellular homeostasis may be a promising strategy for the treatment of inflammatory and autoimmune diseases [12,13].

The L-arginine/NO pathway is involved in maintaining homeostasis in the human body and its alteration has important implications for various physiological processes in the skin [14]. Under inflammatory conditions post-translational changes in L-arginine, take place resulting in the synthesis of ADMA, SDMA and MMA. Once ADMA and MMA are generated, they compete with L-arginine for binding to the active site of NO synthase, which reduces the production of NO. Additionally, SDMA inhibits the transport of L-arginine, leading to a lower concentration of this amino acid within cells. Consequently, by impacting both the availability of L-arginine and the production of NO, conditions favoring oxidative stress and endothelial dysfunction are created [14].

Chronic inflammatory skin diseases constitute a heterogeneous group of conditions with complex pathogenesis that, in most cases, has not been fully elucidated. These disorders are characterised by abnormal immune responses triggered by various stimuli represented by exogenous or endogenous factors, which in turn activate numerous signalling pathways in the skin. These cascade events lead to a chronic inflammatory state, which over time becomes persistent, resulting in a variety of pathological changes. Psoriasis, atopic dermatitis, and vitiligo are among the most common chronic inflammatory skin diseases. Although they have been intensively studied in recent years, many unknowns remain regarding their pathogenesis [15,16,17]. Recent studies have focused on the role of methylarginines in skin diseases. Little is known in this area, but the L-arginine/NO pathway could open up new perspectives in the pathogenesis of these diseases. This review aims to comprehensively synthesize the current evidence regarding methylarginine levels in chronic inflammatory skin diseases, focusing on their potential role as mediators of inflammatory and oxidative stress pathways. By analysing and integrating findings from existing studies, our objective is to provide new insights into the mechanisms underlying these conditions. This can serve as a basis for developing novel diagnostic biomarkers and innovative therapeutic strategies targeting these molecular pathways. To the best of our knowledge, this is the first review that brings together all articles published on this topic.

## 2. Materials and Methods

We conducted a narrative review using the following databases: PubMed, Google Scholar, Web of Science, Scopus, and ScienceDirect. We used as keywords SDMA, ADMA, MMA, methylarginine, inflammatory skin diseases. We included original articles that evaluated methylarginine levels in patients with inflammatory skin diseases. We excluded reviews, abstracts, clinical cases, and articles that evaluated methylarginine levels in skin conditions other than chronic inflammatory skin diseases. After applying the inclusion and exclusion criteria, we analyzed 15 original articles. Due to the limited number of available articles and the considerable heterogeneity in their design, methodology, and reported outcomes, it was not feasible to conduct either a meta-analysis or a systematic review. Narrative reviews are useful when systematic reviews are impractical. These reviews are more accessible for clinicians and are valuable for highlighting gaps in knowledge.

In this article, we did not include articles that evaluated methylarginine levels in patients with rheumatic diseases associated with skin manifestations such as systemic sclerosis, systemic lupus erythematosus, Behcet’s disease, etc. In this regard, a meta-analysis was conducted in 2019 that analyzed methylarginine levels in patients with rheumatic diseases. The meta-analysis included a significant number of participants (1860 patients with rheumatic diseases and 1122 healthy subjects). Most studies demonstrated a link between elevated methylarginine levels and rheumatic diseases, but some studies did not identify this association. The authors attribute the conflicting results to the limited sample sizes included in some of the studies [18].

**Figure 1:**
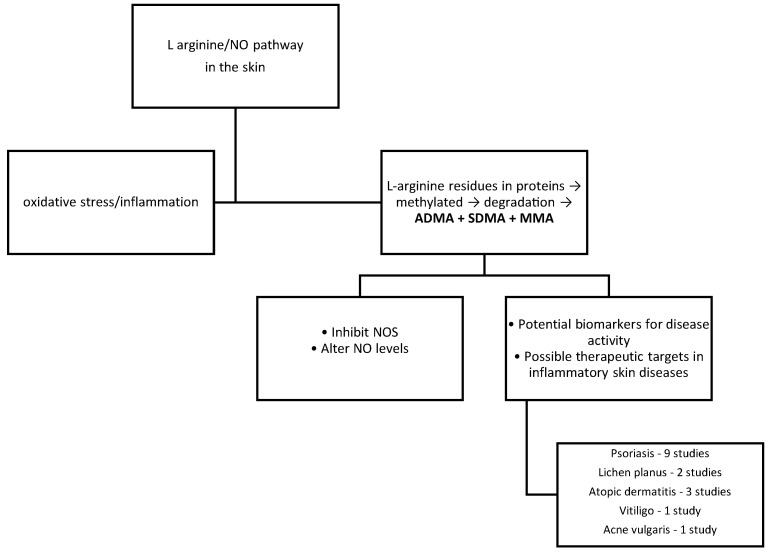
Schematic representation of the mai points of this review. NO—nitric oxide; NOS—nitric oxide synthase; MMA—monomethylarginine; ADMA—asymmetric dimethylarginine; SDMA—symmetric dimethylarginine.

The chronic inflammatory skin diseases in which methylarginine levels were studied are psoriasis, atopic dermatitis, lichen planus, vitiligo, and acne. Figure 1 presents a summary of the studies included in this review. A total of 9 out of 15 studies analyzed patients with psoriasis. In the following sections we present the results of these studies.

## 3. Methylarginines in Chronic Inflammatory Skin Diseases

ADMA and SDMA are methylarginines resulting from post-translational modifications of proteins, namely the methylation of L-arginine incorporated into proteins [19]. The two compounds were first identified in urine. To date, nine enzymes involved in this process, known as protein PRMTs, have been identified. These enzymes can be classified into three classes, leading to the formation of different methylarginines: NG monomethyl-L-arginine (L-NMMA) and dimethylarginines, asymmetric dimethylarginine (ADMA) and symmetric dimethylarginine (SDMA). Subsequently, through proteolysis, free ADMA and SDMA are released into the cell cytoplasm and transported by a cationic amino acid transporter (CAT), a membrane protein, to various organs [20,21,22]. Over time, studies have focused more on ADMA than on SDMA, the latter being considered an isomer of ADMA and a biologically inert compound. ADMA has been described as a multifaceted molecule, but its main role is to inhibit NOS [23]. In addition, it has been shown that ADMA can interfere with various signaling pathways (chemokines, NOD-like receptors, toll-like receptors, MAPK), modulate the interaction between cytokines and specific receptors, and regulate certain metabolic pathways such as those of arachidonic acid or tyrosine. Abnormal ADMA levels promote aberrant expression of several genes, especially genes expressed by endothelial cells. It should be noted that ADMA exerts effects not only through NOS inhibition but also via other pathways, which are not yet elucidated. Less is known about the effects of SDMA. It should be noted that, like ADMA, it is a down-regulator of NO production, but it also exerts oxidative and pro-inflammatory effects. SDMA is involved in inducing the expression of certain receptors on inflammatory cells such as polymorphonuclear cells or monocytes to potentiate the inflammatory process. NMMA is a little-studied molecule, but what is known is that it is an inhibitor of NOS [20,21].

ADMA is excreted renally in a proportion of up to 10%. In contrast, SDMA is eliminated by glomerular filtration in a percentage of over 90%. ADMA is metabolised into two main compounds, citrulline and dimethylamine, in reactions catalysed by dimethylarginine dimethylaminohydrolases (DDAHs) [24]. ADMA metabolism occurs in pancreatic and renal cells, as well as in immune cells. Another enzyme involved in ADMA metabolism is alanine-glyoxylate aminotransferase 2 (AGXT2) [25]. The activity of the enzymes responsible for ADMA metabolism can be influenced by various factors, including an imbalance between oxidants and prooxidants [22]. Under conditions of increased inflammation and oxidative stress, there is an upregulation of arginine methyltransferases, downregulation of DDAHs and increased arginase activity, a combination of processes associated with the synthesis of methylarginine and ornithine [3,26,27]. There is a vicious cycle. Thus, increased levels of dimethylarginine lead to the generation of oxidative stress, but at the same time, under conditions of oxidative stress, there is increased production of dimethylarginines. It has been shown that at the endothelial level, ADMA triggers oxidative stress by inhibiting NO synthesis. The link between dimethylarginine overproduction and oxidative stress is also reflected in the results of a study that showed that after the administration of acetylsalicylic acid, ADMA levels decreased as a result of the anti-inflammatory and protective effect of acetylsalicylic acid against oxidising molecules [28].

Significant progress has been made in understanding the pathogenesis of chronic inflammatory skin diseases, and new therapies have been developed. However, there are still many unknowns. Two key processes involved in the development of these disorders are inflammation and oxidative stress. Therefore, it is crucial to understand the triggers of these pathogenic pathways and the molecules involved. Current research suggests that methylarginines could serve as markers for inflammation and oxidative stress. As a result, these compounds may be used to evaluate pathological processes in patients with skin conditions. Additionally, understanding the dysfunction of specific pathways could lead to the development of new therapies. In cases where the L-arginine/NO pathway is altered, there may be a therapeutic opportunity to utilize NO. Studies have shown that products that release NO can lead to improvements in disorders such as atopic dermatitis [29]. Moreover, a recent study involving patients with acne vulgaris showed that applying a NO-producing gel resulted in reduced inflammatory signs and fewer pustules [30].

### 3.1. Psoriasis

In nine studies, the levels of methylarginines were evaluated, in seven of these, measurements were conducted in serum, while in two, assessments were performed on skin tissue (Table 1). In most studies, statistically significantly higher levels of ADMA were identified in patients with psoriasis compared to the control group. In this regard, for example, Bilgiç et al. found significantly higher serum levels of ADMA and also reported a positive correlation between serum levels of ADMA and psoriasis severity, suggesting that ADMA may serve as a potential biomarker for disease severity. The study cohort included patients with moderate to severe psoriasis, with a mean Psoriasis Area and Severity Index (PASI) score of 10.6. In contrast, another study reported a non-significant elevation in serum ADMA levels in patients with psoriasis compared to the control group. This finding may be attributed to the inclusion of patients with mild to moderate disease severity, with a mean PASI score of 5.3 [31]. Similarly, Usta et al. reported statistically non-significant results in their study, which included patients with a mean PASI score of 4.6 [32]. Another interesting finding is that patients who had higher ADMA levels before starting adalimumab therapy at 6 months after therapy achieved better therapeutic responses. Similarly, in patients with psoriatic arthritis, normalization of the L-arginine/ADMA ratio was observed after treatment with anti-TNF alpha agents [33]. Only one study evaluated SDMA and L-NMMA levels and did not find significant differences between groups.

NO levels influence keratinocyte behaviour; therefore, when found in increased amounts, it acts as an inhibitor of proliferation, whereas low levels lead to increased growth of keratinocytes. Although many unknowns remain regarding the link between ADMA and oxidative stress, it is clear that under conditions of oxidative stress, serum ADMA levels increase. In fact, increased serum ADMA levels are an indicator of vascular oxidative stress, which in turn reflects increased systemic oxidative stress. Under conditions of oxidative stress, both the synthesis and degradation of ADMA are inhibited [34]. Göçer Gürok et al. identified a statistically significant negative correlation between serum ADMA levels and glutathione, an important antioxidant, in patients with psoriasis [34].

There are numerous studies supporting the involvement of oxidative stress in the pathogenesis of psoriasis [35,36,37]. When there is a deficiency of the L-arginine substrate and increased amounts of ADMA at the cellular level, the activity of endothelial NOS is disturbed, which is the basis for the onset of oxidative stress. In turn, reactive oxygen species exert an inhibitory effect on DDAH, the main enzymes involved in ADMA catabolism, resulting in a further increase in ADMA levels [38]. SDMA acts as a compound that inhibits NO production. The main mechanism by which it exerts this effect is by inhibiting the transport of cationic amino acids that are necessary for L-arginine to enter the cell and for L-arginine absorption at the renal level. SDMA promotes the generation of reactive oxygen species by increasing calcium entry into monocytes and activating NADPH oxidase [20]. In addition, SDMA can act at the endothelial level as an oxidising factor, increasing the synthesis of reactive oxygen species, which decreases arginine absorption at the cellular level and, implicitly, NO production [22].

Psoriasis is a chronic immune-mediated inflammatory disease, that mainly affects the skin. It is now accepted that, in addition to the inflammatory process that occurs in the skin, patients with psoriasis also experience systemic inflammation that contributes to significant immune abnormalities leading to pathologies such as diabetes, cardiovascular disease and metabolic disorders, which are frequently diagnosed in these patients [39,40]. Several studies have shown that ADMA may be an indicator of the risk of cardiovascular disease and atherosclerosis [41,42]. It should be noted that these conditions are more common in patients with psoriasis. Ilves et al. identified elevated ADMA levels and a reduced ornithine-to-arginine ratio in patients with psoriasis, suggesting disruptions in the urea cycle, which may reflect high nucleic acid metabolism associated with increased cell proliferation [43]. Atzeni et al. identified elevated serum ADMA levels in patients with psoriatic arthritis and suggested that ADMA could be considered a marker of endothelial dysfunction [44]. Inflammatory cells and the cytokines they release are the main factors contributing to the development of psoriatic lesions and endothelial cell dysfunction. Among the cytokines released, IL-6, IL-17, TNF alpha, and IFN gamma in particular contribute to the development of endothelial dysfunction [45].

**Table 1:** Methylarginine levels in patients with psoriasis.

Disease	Study Participants	Study Type	PASI	Parameter (Patients Versus Controls)	Sample	Conclusion	Reference
**Psoriasis**	59 patients 40 controls	Case–control.	Not available	ADMA—higher (1.15 ± 0.43 vs. 0.76 ± 0.39 µmol/L)	Serum	ADMA plays an important role in the pathogenesis of psoriasis, and the development of therapies aimed at lowering its levels could open new perspectives in psoriasis treatment.	Göçer Gürok et al. (2025) [34]
**Psoriasis** **Atopic dermatitis**	20 psoriasis patients 15 atopic dermatitis patients	Case–control	7.7 ± 2.3	ADMA—higher in psoriasis patients (0.4483 vs. 0.1622 µmol/L)	Skin	These differences suggest the different pathogenic mechanisms underlying the occurrence of the 2 diseases	Ilves et al. (2021) [46]
**Psoriasis**	20 patients 19 controls	Case–control	Not available	ADMA—higher (lesional skin vs. non-lesional skin) (1.419 vs. 0.475 µmol/L) ADMA—higher (lesional skin vs. control) (1.419 vs. 0.571 µmol/L)	Lesional skin Healthy skin (from psoriasis patients) Control skin (from controls)	The accumulation of ADMA in the skin of patients with psoriasis indicates its role in disease pathogenesis	Pohla et al. (2020) [47]
**Psoriasis**	29 patients with psoriasis before treatment with adalimumab 29 patients with psoriasis after treatment with adalimumab	Prospective cohort study	18.9 ± 7.8	ADMA -correlated with BSA (1.523 vs. 0.403 µmol/L + before and after treatment with adalimumab)	Serum	ADMA may be a marker of disease severity in patients with psoriasis and a predictor of the response to treatment	Pina et al. (2016) [33]
**Psoriasis**	40 patients 40 controls	Case–control	Not available	ADMA—higher (1.49 ± 0.09 vs. 0.46 ± 0.06 µmol/L)	Serum	ADMA can be considered a marker of disease severity, given that in patients with severe forms the levels were higher than in those with mild forms.	Abdul Kareem et al. (2016) [48]
**Psoriasis**	40 patients 40 controls	Case–control	5.32 ± 4.09	ADMA—NS (0.19 ± 0.06 vs. 0.17 ± 0.084 µmol/L)	Serum	The role of ADMA is unclear in the pathogenesis of psoriasis; further studies are needed.	Bilgiç et al. (2016) [49]
**Psoriasis**	42 patients 48 controls	Case–control	10.64 ± 6.77	ADMA—higher (1.08 ± 0.23 vs. 0.60 ± 0.25 µmol/L) SDMA—NS (1.07 ± 0.29 vs. 0.94 ± 0.47 µmol/L) L-NMMA—NS (0.13 ± 0.14 vs. 0.66 ± 0.79 µmol/L)	Serum	Among L-arginine/NO pathway metabolites, ADMA appears to hold the most important role in the pathogenesis of psoriasis.	Bilgiç et al. (2015) [31]
**Psoriasis**	35 patients 26 controls	Case–control	4.6 ± 5.7	ADMA-NS (0.63 ± 0.30 vs. 0.68 ± 0.65 µmol/L)	Serum	ADMA does not represent an indicator of endothelial disfunction in psoriasis patients	Turan et al. (2014) [50]
**Psoriasis**	29 patients 25 controls	Case–control	4.6 ± 3.8	ADMA—NS (0.44 ± 0.06 vs. 0.46 ± 0.07 µmol/L)	Serum	In patients with mild/moderate forms of psoriasis associated with mild inflammation, serum ADMA levels do not increase.	Usta et al. (2011) [32]

ADMA—asymmetric dimethylarginine, SDMA—symmetric dimethylarginine, L-NMMA—LN-monomethyl-L-arginine, BSA—body surface area, NS—no significant differences.

### 3.2. Vitiligo

We have found only one study that evaluated the serum levels of ADMA in vitiligo patients (Table 2). Vitiligo is an autoimmune depigmenting skin disease characterised by an important inflammatory process [51]. Emerging evidence suggests that certain local factors can stimulate innate immune cells and contribute to the onset of the disease before adaptive immunity is engaged to target melanocytes. This process is similar to that observed in inflammatory skin diseases such as psoriasis and atopic dermatitis [52]. In patients with vitiligo, increased levels of ADMA were found in the context of an imbalance between oxidants and antioxidants, with significantly lower levels of vitamin E, an important antioxidant, and significantly higher levels of malondialdehyde, one of the most important products resulting from lipid peroxidation, compared to the control group, confirming the fact that the alteration of the L-arginine/NO pathway occurs under conditions of oxidative stress [53].

### 3.3. Atopic Dermatitis

Atopic dermatitis is a chronic inflammatory skin condition with a complex pathogenesis in which skin barrier dysfunction and immunological abnormalities play a cardinal role. Genetic and environmental factors are crucial in the onset and evolution of atopic dermatitis [54,55]. Endothelial dysfunction has also been highlighted in these patients accompanied by increased endothelial levels of VECAM and VEGF. It has been found that NOS is responsible for the microvascularisation of the upper dermis and contributes to the development of both inflammatory and non-inflammatory lesions in atopic dermatitis [56]. Of the 3 studies that evaluated the levels of patients with atopic dermatitis, in one of them, there were no significant differences compared to the control group, in one of them ADMA levels were higher in lesional skin and in the 3rd, there were higher levels of ADMA compared to patients with psoriasis (Table 1 and Table 2).

NO has been shown to play a role in the pathogenesis of atopic diseases; therefore studying the L-arginine/NO pathway may yield interesting findings. It should be noted that there is a competition between ADMA and arginine to bind to NOS. The Arg/ADMA and Arg/SDMA ratios are markers for L-arginine availability; therefore, low levels of these ratios are associated with low L-arginine availability, which is essential for NO production [22]. As the Arg/ADMA ratio was also significantly reduced in atopic patients, it suggests that NOS activity may be inhibited in these patients. Thus, a lower Arg/ADMA ratio can be seen as a potential feedback mechanism to regulate NOS activity. In addition, increased levels of DMA, one of the main metabolites of ADMA, have been observed in patients with atopic diseases, which may be an indirect sign of increased ADMA levels [57].

### 3.4. Lichen Planus

Cutaneous lichen planus is an inflammatory skin disease predominantly driven by T lymphocytes [58]. The pathogenesis of the disease lies at the intersection of genetic factors, immune/inflammatory response abnormalities, oxidative stress, and environmental and psychological factors [59]. There are two studies available in the medical literature that have investigated serum SDMA levels in patients with lichen planus, and both studies identified significantly higher SDMA levels in patients compared to controls. In lichen planus, SDMA appears to be a marker indicating an imbalance of oxidants and pro-oxidants, as well as a pro-inflammatory status (Table 2). Thus, in patients with cutaneous lichen planus, Tampa et al. identified a positive correlation with serum hsCRP levels and a negative correlation with total antioxidant status [60].

### 3.5. Acne Vulgaris

A single study evaluated the levels of methylarginines, ADMA, SDMA, and L-NMMA in patients with acne vulgaris (Table 2). Four main factors are involved in the appearance of acne lesions, namely sebum secretion, *Cutibacterium* acnes colonisation, hormones, and the keratinization process [61,62]. As discussed above, inflammation and oxidative stress are inducers for the generation of methylarginines. In patients with acne, *C. acnes* plays a defining role in the initiation of inflammation by releasing chemotactic factors for neutrophils, which leads to their accumulation in acne lesions. Neutrophils, in turn, release reactive oxygen species, which cause tissue damage, a phenomenon called “auto-oxidative damage” [63]. Akyurek et al. highlighted that there is a directly proportional relationship between methylarginine levels and disease severity in patients with acne. A direct relationship was also observed between disease severity and oxidative stress levels assessed by ischemia modified albumin (IMA), a marker that has been intensively studied recently and appears to be an important indicator of oxidative stress and not just ischemia, as previously described [63]. Although ADMA and L-NMMA have an inhibitory role on NOS, increased levels of L-arginine will lead to the unblocking of NO synthesis, a phenomenon known as the arginine paradox [63]. Kumtornrut et al. developed a cleanser containing Tris (hydroxymethyl) aminomethane and L-arginine which improved acne lesions. The product proved effective in regulating sebum secretion and keratin plug formation [64].

**Table 2:** Methylarginine levels in patients with chronic inflammatory skin diseases other than psoriasis.

Disease	Study Participants	Study Type	Clinical Characteristics	Parameter (Patients Versus Controls)	Sample	Conclusion	Reference
**Lichen planus**	40 patients 40 controls	Case–control	Classic lichen planus—31 patients Hypertrophic lichen planus—6 patients Annular lichen planus—3 patients Oral lichen planus—patients	SDMA—higher (0.82 ± 0.20 vs. 0.49 ± 0.06 µmol/L)	Serum	Nitrosation stress may play a role in the pathogenesis of cutaneous lichen planus.	Tampa et al. (2024) [60]
**Atopic diseases** **(bronchial asthma, atopic dermatitis)**	81 patients 30 controls (individuals without allergy)	Case–control	Moderate atopic dermatitis—36 patients Severe atopic dermatitis—9 patients	ADMA—NS (plasma) (0.68 vs. 0.65 µmol/L) ADMA—NS (urine) (6.17 vs. 7.24 µmol/L) SDMA—NS (urine) (6.71 vs. 6.59 µmol/L)	Plasma, urine	L-arginine/NO pathway abnormalities in patients with atopic diseases do not correlate with disease severity	Hanusch et al. (2022) [57]
**Atopic dermatitis**	17 patients	Case–control	Not available	ADMA—higher (lesional skin vs. non-lesional skin) (0.519 vs. 0.262 µmol/L)	Lesional skin Healthy skin (from atopic dermatitis patients)	ADMA could serve as a biomarker of inflammation and metabolic dysregulation in atopic dermatitis.	Ilves et al. (2022) [43]
**Lichen planus**	31 patients 26 controls	Case–control	Cutaneous lichen planus—31 patients Oral lichen planus—5 patients Lichen planopilaris—1 patient Nail lichen planus—3 patients	SDMA—higher (0.84 ± 0.19 vs. 0.50 ± 0.06 µmol/L)	Serum	SDMA may represent a potential marker of oxidative stress in patients with lichen planus.	Mitran et al. (2021) [65]
**Acne vulgaris**	90 patients 30 controls	Case–control	Mild acne—30 patients Moderate acne—30 patients Severe acne—30 patients	ADMA—higher (0.48 ± 0.15 vs. 0.37 ± 0.12 µmol/L) SDMA—higher (0.48 ± 0.19 vs. 0.41 ± 0.12 µmol/L) L-NMMA—higher (0.07 ± 0.03 vs. 0.06 ± 0.02 µmol/L)	Plasma	L-arginine/NO pathway is involved in the pathogenic processes observed in acne and influences the course of the disease	Tunçez Akyürek et al. (2020) [63]
**Vitiligo**	30 patients 20 controls	Case–control	Not available	ADMA—higher (0.49 ± 0.2 vs. 0.32.0.1 µmol/L)	Serum	Increased levels of ADMA may be involved in the pathogenesis of vitiligo	Kaman et al. (2016) [53]

ADMA—asymmetric dimethylarginine, SDMA—symmetric dimethylarginine, NS—no significant differences.

## 4. Methylarginines in Other Skin Diseases

We consider it worth mentioning other studies that have evaluated methylarginine levels in skin conditions other than inflammatory skin diseases. Therefore, methylarginines have also been studied in allergic conditions such as acute urticaria, skin tumors (melanoma) and skin infections caused by *Bacillus anthracis*.

Given that the pathogenesis of melanoma is complex and incompletely elucidated [66,67], a recent study evaluated the serum levels of methylarginines and the ADMA/SDMA ratio in patients with cutaneous melanoma. Higher levels of ADMA and SDMA were found in cancer patients compared to the control group. The authors concluded that dysregulation of NO pathway is associated with the generation of nitrosative stress and abnormalities in metabolic pathways involved in tumor progression [68]. Research shows that elevated ADMA levels alter macrophage migration, interfere with phagocytosis, and induce M2 phenotype dominance; all these events can facilitate metastasis. Tumor cells secrete elevated levels of ADMA, and from a genetic perspective, there is an increased expression of genes involved in the production of ADMA to the detriment of those mediating the metabolism of this compound [69,70]. Sunnetcioglu et al. studied ADMA levels in patients with cutaneous anthrax and identified significantly higher levels among those infected compared to the control group. In addition, a direct correlation was observed between ADMA levels and the degree of inflammation assessed by erythrocyte sedimentation rate. These results could be explained by the presence of perivascular inflammatory infiltrate and vasculitis observed in cutaneous anthrax lesions [71]. Based on the fact that ADMA serves as an indicator of elevated vascular resistance, a factor that contributes to the development of urticaria, Tuncer et al. studied this marker in patients with acute urticaria. They included 77 patients with acute urticaria and evaluated plasma ADMA variations during and after an episode of urticaria. However, the results were statistically insignificant [72].

The studies analyzed in this review show that methylarginines may play a role in the pathogenesis of chronic inflammatory skin diseases, but at the same time, we should mention that the number of studies analyzed was limited, the studies included small groups of patients, and the biological products used for the measurement were heterogeneous, including plasma, serum, urine, or skin tissue. Consequently, further studies are needed to consolidate our understanding of the impact of methylarginine on the pathogenic processes in inflammatory skin diseases and to form the basis for research targeting molecules that modulate the L-arginine/NO pathway, which may represent potential new therapeutic targets.

## 5. Future Research Directions

The role of L-arginine is more complex than previously understood, as it is central to various translational processes that are correlated with genetic and epigenetic programs. Its involvement in genetic mutations and epigenetic modifications can significantly impact inflammatory states within immune cells. Under normal physiological conditions, the levels of l-arginine are regulated by arginine-tRNA synthetase (ArgRS) and serine/arginine repetitive matrix protein 2 (SRRM2). SRRM2, which has splicing functions, associates with ArgRS in the nucleus [73].

During inflammation, L-arginine levels decrease, leading to a reduction in nuclear ArgRS. This change enhances the splicing function of SRRM2, resulting in significant modifications to the cellular proteome. In this inflammatory context, alternative splice variants of fibroblast growth factor receptor 3 (FGFR3) and pleckstrin homology domain-containing family G member 5 (PLEKHG5) are generated. The alternative splicing of FGFR3 can result in hyperactivation and autocrine stimulation of the cells [74,75,76].

At the genetic level, the depletion of l-arginine reduces the levels of arginine-tRNAs, which delays ribosome movement over arginine codons. This mechanistic process stalls the translation of arginine codons, leading to a shift in the proteome towards genes with fewer arginine codons. Over the course of chronic inflammation, this pressure may induce mutations and select immune cells with specific proteomic characteristics that support the ongoing inflammatory response [77].

The mechanistic target of rapamycin complex 1 (mTORC1) is sensitive to cellular nutrients and serves as the master regulator of metabolism, with amino acids controlling its recruitment and activation. In conditions of low nutrient availability, including arginine, the GTPase-activating protein complex GATOR1 promotes GTP hydrolysis on RagA/B, resulting in the inactivation of mTORC1. As a consequence of l-arginine deficiency, the levels of H3K27 acetylation on genes, such as Tbx21 and Gata3 (which are essential for T cell activation), decrease significantly. Given the crucial role of mTORC1 activation in coordinating inflammatory processes, recent research emphasizes the importance of the ArgRS and mTORC1 pathways in sensing l-arginine levels during inflammation [78,79].

Future investigations into l-arginine pathways in relation to skin diseases should focus on adjustments in metabolic pathways, as dysbiosis may provide a potential therapeutic reservoir for sustained clinical management and quality of life improvement. At the level of keratinocytes, continuously expressed l-arginine transporters, specifically Cationic Amino Acid Transporter-1 (CAT1) and -2 (CAT2), facilitate l-arginine metabolism to nitric oxide (NO), serving as an important checkpoint for l-arginine levels in the bloodstream. In psoriatic patients, reduced blood levels of l-arginine may be associated with increased levels of arginase in psoriatic lesions, suggesting a role for CAT1 and CAT2 in managing psoriasis. Furthermore, changes in amino acids within the skin microenvironment could serve as emerging biomarkers for psoriasis and may indicate early metabolic imbalances related to the disease [80,81].

Since systemic inflammation and oxidative stress play pivotal roles in the pathogenesis of various skin diseases, particularly psoriasis, therapeutic strategies aimed at reducing oxidative stress may greatly benefit patient health. Therefore, future scientific research should involve larger populations and include comprehensive assessments of oxidative stress parameters. Upcoming clinical trials should be designed to evaluate the reduction in asymmetric dimethylarginine (ADMA) and symmetric dimethylarginine (SDMA) levels through pharmacological agents, thereby defining new therapeutic targets and effective strategies for managing dermatological conditions [34].

Excessive methylarginine production is closely associated with processes such as inflammation, immunity, and oxidative stress. As discussed above, PRMTs are essential enzymes in the methylarginine generation pathway. Therefore, compounds that inhibit the activity of PRMTs may represent new therapeutic approaches for inflammatory skin diseases. One such compound, TC-E 5003, specifically blocks the activity of PRMT1 and has demonstrated anti-inflammatory effects by acting on TLR4. Additionally, this compound downregulates LPS-mediated NO production and reduces the expression of pro-inflammatory molecules, including IL-6 and TNF-alpha. These molecules play a crucial role in the development of inflammatory skin conditions, such as psoriasis [12,82].

In this review, we primarily focused on studying methylarginine levels in patients with chronic inflammatory skin diseases. In the future, we intend to study the roles of PRMTs and the potential effects of their inhibition. Additionally, we consider it would be useful to review the literature concerning the relationship between methylarginine and other markers of oxidative stress.

## 6. Conclusions

The L-arginine/NO pathway is essential for skin homeostasis, and its disruption is closely linked to inflammation and oxidative stress. Thus, in the presence of pro-inflammatory molecules and oxidants, excessive production of methylarginine occurs, particularly ADMA, which is also the most studied compound in this regard. High amounts of dimethylarginines alter keratinocyte behaviour, interfere with skin signalling pathways and maintain elevated levels of oxidative stress. Among chronic inflammatory skin diseases, methylarginines have been studied, particularly in psoriasis, where the results are contradictory, most likely influenced by the severity of the disease. In other chronic inflammatory skin diseases, studies are few, making it difficult to conclude, but we believe that further investigation is warranted to consolidate our knowledge of the role of methylarginines in the pathogenesis of these conditions.

## References

[1] Caldwell R.W., Rodriguez P.C., Toque H.A., Narayanan S.P., Caldwell R.B. (2018). Arginase: A Multifaceted Enzyme Important in Health and Disease. Physiol. Rev..

[2] Farahani A., Farahani A., Kashfi K., Ghasemi A. (2025). Inducible Nitric Oxide Synthase (iNOS): More than an Inducible Enzyme? Rethinking the Classification of NOS Isoforms. Pharmacol. Res..

[3] González M., Rivas J.C., González M., Rivas J.C. (2020). L-Arginine/Nitric Oxide Pathway and KCa Channels in Endothelial Cells: A Mini-Review. Vascular Biology—Selection of Mechanisms and Clinical Applications.

[4] Kim J.H., Choi M.S. (2023). Nitric Oxide Signal Transduction and Its Role in Skin Sensitization. Biomol. Ther..

[5] Liy P.M., Puzi N.N.A., Jose S., Vidyadaran S. (2021). Nitric Oxide Modulation in Neuroinflammation and the Role of Mesenchymal Stem Cells. Exp. Biol. Med..

[6] Andrabi S.M., Sharma N.S., Karan A., Shahriar S.M.S., Cordon B., Ma B., Xie J. (2023). Nitric Oxide: Physiological Functions, Delivery, and Biomedical Applications. Adv. Sci..

[7] Hall E.T., Fernandez-Lopez E., Silk A.W., Dummer R., Bhatia S. (2020). Immunologic Characteristics of Nonmelanoma Skin Cancers: Implications for Immunotherapy. Am. Soc. Clin. Oncol. Educ. Book.

[8] Lundberg J.O., Weitzberg E. (2022). Nitric Oxide Signaling in Health and Disease. Cell.

[9] Zaborova V., Budanova E., Kryuchkova K., Rybakov V., Shestakov D., Isaikin A., Romanov D., Churyukanov M., Vakhnina N., Zakharov V. (2025). Nitric Oxide: A Gas Transmitter in Healthy and Diseased Skin. Med. Gas Res..

[10] Matei C., Tampa M., Caruntu C., Ion R.-M., Georgescu S.-R., Dumitrascu G.R., Constantin C., Neagu M. (2014). Protein Microarray for Complex Apoptosis Monitoring of Dysplastic Oral Keratinocytes in Experimental Photodynamic Therapy. Biol. Res..

[11] Gayatri S., Bedford M.T. (2014). Readers of Histone Methylarginine Marks. Biochim. Biophys. Acta BBA Gene Regul. Mech..

[12] Srour N., Khan S., Richard S. (2022). The Influence of Arginine Methylation in Immunity and Inflammation. J. Inflamm. Res..

[13] Guo Q., Jin Y., Chen X., Ye X., Shen X., Lin M., Zeng C., Zhou T., Zhang J. (2024). NF-κB in Biology and Targeted Therapy: New Insights and Translational Implications. Signal Transduct. Target. Ther..

[14] Stancic A., Jankovic A., Korac A., Buzadzic B., Otasevic V., Korac B. (2018). The Role of Nitric Oxide in Diabetic Skin (Patho)Physiology. Mech. Ageing Dev..

[15] Liu Y., Wang H., Taylor M., Cook C., Martínez-Berdeja A., North J.P., Harirchian P., Hailer A.A., Zhao Z., Ghadially R. (2022). Classification of Human Chronic Inflammatory Skin Disease Based on Single-Cell Immune Profiling. Sci. Immunol..

[16] Litman T. (2019). Personalized Medicine—Concepts, Technologies, and Applications in Inflammatory Skin Diseases. APMIS.

[17] Ho A.W., Kupper T.S. (2019). T Cells and the Skin: From Protective Immunity to Inflammatory Skin Disorders. Nat. Rev. Immunol..

[18] Erre G.L., Mangoni A.A., Castagna F., Paliogiannis P., Carru C., Passiu G., Zinellu A. (2019). Meta-Analysis of Asymmetric Dimethylarginine Concentrations in Rheumatic Diseases. Sci. Rep..

[19] Jarzebska N., Mangoni A.A., Martens-Lobenhoffer J., Bode-Böger S.M., Rodionov R.N. (2019). The Second Life of Methylarginines as Cardiovascular Targets. Int. J. Mol. Sci..

[20] Tain Y., Hsu C. (2017). Toxic Dimethylarginines: Asymmetric Dimethylarginine (ADMA) and Symmetric Dimethylarginine (SDMA). Toxins.

[21] Thiebaut C., Eve L., Poulard C., Le Romancer M. (2021). Structure, Activity, and Function of PRMT1. Life.

[22] Nicolae I., Tampa M., Grigore M., Mitran C.I., Mitran M.I., Dulgheru L., Georgescu S.R. (2020). Symmetrical Dimethylarginine (Sdma)And Venous Ulcer Of THE Lower Limbs. Dermatovenerol. J..

[23] Schwedhelm E., Böger R.H. (2011). The Role of Asymmetric and Symmetric Dimethylarginines in Renal Disease. Nat. Rev. Nephrol..

[24] Vallance P., Leiper J. (2004). Cardiovascular Biology of the Asymmetric Dimethylarginine: Dimethylarginine Dimethylaminohydrolase Pathway. Arterioscler. Thromb. Vasc. Biol..

[25] Rodionov R.N., Jarzebska N., Burdin D., Todorov V., Martens-Lobenhoffer J., Hofmann A., Kolouschek A., Cordasic N., Jacobi J., Rubets E. (2022). Overexpression of Alanine-Glyoxylate Aminotransferase 2 Protects from Asymmetric Dimethylarginine-Induced Endothelial Dysfunction and Aortic Remodeling. Sci. Rep..

[26] Cziráki A., Lenkey Z., Sulyok E., Szokodi I., Koller A. (2020). L-Arginine-Nitric Oxide-Asymmetric Dimethylarginine Pathway and the Coronary Circulation: Translation of Basic Science Results to Clinical Practice. Front. Pharmacol..

[27] Krzystek-Korpacka M., Szczęśniak-Sięga B., Szczuka I., Fortuna P., Zawadzki M., Kubiak A., Mierzchała-Pasierb M., Fleszar M.G., Lewandowski Ł., Serek P. (2020). L-Arginine/Nitric Oxide Pathway Is Altered in Colorectal Cancer and Can Be Modulated by Novel Derivatives from Oxicam Class of Non-Steroidal Anti-Inflammatory Drugs. Cancers.

[28] Aydin M., Koca C., Uysal S., Totan Y., Yağci R., Armutcu F., Cücen Z., YiĞiToğlu M.R. (2012). Serum Nitric Oxide, Asymmetric Dimethylarginine, and Plasma Homocysteine Levels in Active Behçet’s Disease. Turk. J. Med. Sci..

[29] Man M.-Q., Wakefield J.S., Mauro T.M., Elias P.M. (2022). Regulatory Role of Nitric Oxide in Cutaneous Inflammation. Inflammation.

[30] Settelmeier S., Rassaf T., Hendgen-Cotta U.B., Stoffels I. (2021). Nitric Oxide Generating Formulation as an Innovative Approach to Topical Skin Care: An Open-Label Pilot Study. Cosmetics.

[31] Bilgiç Ö., Altınyazar H.C., Baran H., Ünlü A. (2015). Serum Homocysteine, Asymmetric Dimethyl Arginine (ADMA) and Other Arginine–NO Pathway Metabolite Levels in Patients with Psoriasis. Arch. Dermatol. Res..

[32] Usta M., Yurdakul S., Aral H., Turan E., Oner E., Inal B.B., Oner F.A., Gurel M.S., Guvenen G. (2011). Vascular Endothelial Function Assessed by a Noninvasive Ultrasound Method and Serum Asymmetric Dimethylarginine Concentrations in Mild-to-Moderate Plaque-Type Psoriatic Patients. Clin. Biochem..

[33] Pina T., Genre F., Lopez-Mejias R., Armesto S., Ubilla B., Mijares V., Dierssen-Sotos T., Corrales A., Gonzalez-Lopez M.A., Gonzalez-Vela M.C. (2016). Asymmetric Dimethylarginine but Not Osteoprotegerin Correlates with Disease Severity in Patients with Moderate-to-severe Psoriasis Undergoing Anti-tumor Necrosis Factor-α Therapy. J. Dermatol..

[34] Göçer Gürok N., Telo S., Genç Ulucan B., Öztürk S. (2025). Oxidative Stress in Psoriasis Vulgaris Patients: Analysis of Asymmetric Dimethylarginine, Malondialdehyde, and Glutathione Levels. Medicina.

[35] Medovic M.V., Jakovljevic V.L., Zivkovic V.I., Jeremic N.S., Jeremic J.N., Bolevich S.B., Ravic Nikolic A.B., Milicic V.M., Srejovic I.M. (2022). Psoriasis between Autoimmunity and Oxidative Stress: Changes Induced by Different Therapeutic Approaches. Oxid. Med. Cell. Longev..

[36] Shakoei S., Nakhjavani M., Mirmiranpoor H., Motlagh M.A., Azizpour A., Abedini R. (2021). The Serum Level of Oxidative Stress and Antioxidant Markers in Patients with Psoriasis: A Cross-sectional Study. J Clin Aesthet Dermatol..

[37] Blagov A., Sukhorukov V., Guo S., Zhang D., Eremin I., Orekhov A. (2023). The Role of Oxidative Stress in the Induction and Development of Psoriasis. Front. Biosci.-Landmark.

[38] Pascale V., Pascale W., Lavanga V., Sansone V., Ferrario P., De Gennaro Colonna V. (2013). L-Arginine, Asymmetric Dimethylarginine, and Symmetric Dimethylarginine in Plasma and Synovial Fluid of Patients with Knee Osteoarthritis. Med. Sci. Monit..

[39] Orlando G., Molon B., Viola A., Alaibac M., Angioni R., Piaserico S. (2022). Psoriasis and Cardiovascular Diseases: An Immune-Mediated Cross Talk?. Front. Immunol..

[40] Gao Y., Xu T., Wang Y., Hu Y., Yin S., Qin Z., Yu H. (2025). Pathophysiology and Treatment of Psoriasis: From Clinical Practice to Basic Research. Pharmaceutics.

[41] Rapone B., Inchingolo F., Tartaglia G.M., De Francesco M., Ferrara E. (2024). Asymmetric Dimethylarginine as a Potential Mediator in the Association between Periodontitis and Cardiovascular Disease: A Systematic Review of Current Evidence. Dent. J..

[42] Sonkar S.K., Verma J., Sonkar G.K., Gupta A., Singh A., Vishwakarma P., Bhosale V. (2025). Assessing the Role of Asymmetric Dimethylarginine in Endothelial Dysfunction: Insights Into Cardiovascular Risk Factors. Cureus.

[43] Ilves L., Ottas A., Kaldvee B., Abram K., Soomets U., Zilmer M., Jaks V., Kingo K. (2022). Metabolomic Differences between the Skin and Blood Sera of Atopic Dermatitis and Psoriasis. Int. J. Mol. Sci..

[44] Atzeni F., Sarzi-Puttini P., Sitia S., Tomasoni L., Gianturco L., Battellino M., Boccassini L., De Gennaro Colonna V., Marchesoni A., Turiel M. (2011). Coronary Flow Reserve and Asymmetric Dimethylarginine Levels: New Measurements for Identifying Subclinical Atherosclerosis in Patients with Psoriatic Arthritis. J. Rheumatol..

[45] Young K.Z., Plazyo O., Gudjonsson J.E. (2023). Targeting Immune Cell Trafficking and Vascular Endothelial Cells in Psoriasis. J. Clin. Investig..

[46] Ilves L., Ottas A., Kaldvee B., Abram K., Soomets U., Zilmer M., Jaks V., Kingo K. (2021). Metabolomic Analysis of Skin Biopsies from Patients with Atopic Dermatitis Reveals Hallmarks of Inflammation, Disrupted Barrier Function and Oxidative Stress. Acta Derm. Venereol..

[47] Pohla L., Ottas A., Kaldvee B., Abram K., Soomets U., Zilmer M., Reemann P., Jaks V., Kingo K. (2020). Hyperproliferation Is the Main Driver of Metabolomic Changes in Psoriasis Lesional Skin. Sci. Rep..

[48] Abdul Kareem I.A., Hamzah M.I., Farhood I.G., Hasan M.M. (2016). Study of Asymmetric Dimethyl Arginine (ADMA) and Anti Cyclic Citrulinated Peptide (Anti-CCP) in Iraqi Patients with Psoriasis Vulgaris. Int. J. Adv. Res..

[49] Bilgiç R., Yıldız H., Karabudak Abuaf Ö., İpçioğlu O.M., Doğan B. (2016). Evaluation of serum asymmetric dimethylarginine levels in patients with psoriasis vulgaris. Turkderm.

[50] Turan H., Arslanyılmaz Z., Bulur S., Acer E., Uslu E., Albayrak H., Aslantaş Y., Memişoğulları R. (2014). Psoriazisli Hastalarda Serum Asimetrik Dimetilarjinin (ADMA) ve Yüksek Sensitif C-Reaktif Protein (Hscrp) Seviyeleri. Düzce Tıp Fakültesi Derg..

[51] Diotallevi F., Gioacchini H., De Simoni E., Marani A., Candelora M., Paolinelli M., Molinelli E., Offidani A., Simonetti O. (2023). Vitiligo, from Pathogenesis to Therapeutic Advances: State of the Art. Int. J. Mol. Sci..

[52] Speeckaert R., Caelenberg E.V., Belpaire A., Speeckaert M.M., Geel N.V. (2024). Vitiligo: From Pathogenesis to Treatment. J. Clin. Med..

[53] Kaman D., DemiR B. (2016). Vitiligolu Hastalarda Serum ADMA, MDA, Vitamin E ve Homosistein Düzeyleri. Fırat Tıp Derg./Firat Med. J..

[54] Schuler C.F., Tsoi L.C., Billi A.C., Harms P.W., Weidinger S., Gudjonsson J.E. (2024). Genetic and Immunological Pathogenesis of Atopic Dermatitis. J. Investig. Dermatol..

[55] Afshari M., Kolackova M., Rosecka M., Čelakovská J., Krejsek J. (2024). Unraveling the Skin; a Comprehensive Review of Atopic Dermatitis, Current Understanding, and Approaches. Front. Immunol..

[56] Steinhoff M., Steinhoff A., Homey B., Luger T.A., Schneider S.W. (2006). Role of Vasculature in Atopic Dermatitis. J. Allergy Clin. Immunol..

[57] Hanusch B., Sinningen K., Brinkmann F., Dillenhöfer S., Frank M., Jöckel K.-H., Koerner-Rettberg C., Holtmann M., Legenbauer T., Langrock C. (2022). Characterization of the L-Arginine/Nitric Oxide Pathway and Oxidative Stress in Pediatric Patients with Atopic Diseases. Int. J. Mol. Sci..

[58] Georgescu S., Tampa M., Mitran M., Mitran C., Sarbu M., Nicolae I., Matei C., Caruntu C., Neagu M., Popa M. (2018). Potential Pathogenic Mechanisms Involved in the Association between Lichen Planus and Hepatitis C Virus Infection (Review). Exp. Ther. Med..

[59] Vičić M., Hlača N., Kaštelan M., Brajac I., Sotošek V., Prpić Massari L. (2023). Comprehensive Insight into Lichen Planus Immunopathogenesis. Int. J. Mol. Sci..

[60] Tampa M., Nicolae I., Ene C.D., Mitran C.I., Mitran M.I., Matei C., Georgescu S.R. (2024). The Interplay between Nitrosative Stress, Inflammation, and Antioxidant Defense in Patients with Lichen Planus. Antioxidants.

[61] Hazarika N. (2021). Acne Vulgaris: New Evidence in Pathogenesis and Future Modalities of Treatment. J. Dermatol. Treat..

[62] Sánchez-Pellicer P., Navarro-Moratalla L., Núñez-Delegido E., Ruzafa-Costas B., Agüera-Santos J., Navarro-López V. (2022). Acne, Microbiome, and Probiotics: The Gut–Skin Axis. Microorganisms.

[63] Tunçez Akyürek F., Saylam Kurtipek G., Kurku H., Akyurek F., Unlu A., Abusoglu S., Ataseven A. (2020). Assessment of ADMA, IMA, and Vitamin A and E Levels in Patients with Acne Vulgaris. J. Cosmet. Dermatol..

[64] Kumtornrut C., Manabe S.D., Navapongsiri M., Okutani Y., Ikegaki S., Tanaka N., Hashimoto H., Songsantiphap C., Wantavornprasert K., Khamthara J. (2020). A Cleanser Formulated with Tris (Hydroxymethyl) Aminomethane and L-arginine Significantly Improves Facial Acne in Male Thai Subjects. J. Cosmet. Dermatol..

[65] Mitran M.I., Tampa M., Nicolae I., Mitran C.I., Matei C., Georgescu S.R., Popa M.I. (2021). New Markers of Oxidative Stress in Lichen Planus and the Influence of Hepatitis C Virus Infection—A Pilot Study. Rom. J. Intern. Med..

[66] Gosman L.M., Țăpoi D.-A., Costache M. (2023). Cutaneous Melanoma: A Review of Multifactorial Pathogenesis, Immunohistochemistry, and Emerging Biomarkers for Early Detection and Management. Int. J. Mol. Sci..

[67] Caruntu C., Mirica A., Roşca A.E., Mirica R., Caruntu A., Tampa M., Matei C., Constantin C., Neagu M., Badarau A.I. (2016). The Role of Estrogens and Estrogen Receptors in Melanoma Development and Progression. Acta Endocrinol..

[68] Ene C.D., Nicolae I. (2022). Hypoxia-Nitric Oxide Axis and the Associated Damage Molecular Pattern in Cutaneous Melanoma. J. Pers. Med..

[69] Chen Y.-L., Lowery A.T., Lin S., Walker A.M., Chen K.-H.E. (2022). Tumor Cell-Derived Asymmetric Dimethylarginine Regulates Macrophage Functions and Polarization. Cancer Cell Int..

[70] Neagu M., Constantin C., Tampa M., Matei C., Lupu A., Manole E., Ion R.-M., Fenga C., Tsatsakis A.M. (2016). Toxicological and Efficacy Assessment of Post-Transition Metal (Indium) Phthalocyanine for Photodynamic Therapy in Neuroblastoma. Oncotarget.

[71] Sunnetcioglu M., Mengeloglu Z., Baran A.I., Karahocagil M., Tosun M., Kucukbayrak A., Ceylan M.R., Akdeniz H., Aypak C. (2014). Asymmetric Dimethylarginine Levels in Patients with Cutaneous Anthrax: A Laboratory Analysis. Ann. Clin. Microbiol. Antimicrob..

[72] Tuncer S.K., Kaldirim Ü., Eyi Y.E., Yildirim A.O., Ekici Ş., Kara K., Eroğlu M., Öztosun M., Özyürek S., Durusu M. (2015). Neopterin, Homocysteine, and ADMA Levels during and after Urticaria Attack. Turk. J. Med. Sci..

[73] Canè S., Geiger R., Bronte V. (2025). The Roles of Arginases and Arginine in Immunity. Nat. Rev. Immunol..

[74] Nofal M., Zhang K., Han S., Rabinowitz J.D. (2017). mTOR Inhibition Restores Amino Acid Balance in Cells Dependent on Catabolism of Extracellular Protein. Mol. Cell.

[75] Cui H., Diedrich J.K., Wu D.C., Lim J.J., Nottingham R.M., Moresco J.J., Yates J.R., Blencowe B.J., Lambowitz A.M., Schimmel P. (2023). Arg-tRNA Synthetase Links Inflammatory Metabolism to RNA Splicing and Nuclear Trafficking via SRRM2. Nat. Cell Biol..

[76] Zhang X., Ibrahimi O.A., Olsen S.K., Umemori H., Mohammadi M., Ornitz D.M. (2006). Receptor Specificity of the Fibroblast Growth Factor Family. The Complete Mammalian FGF Family. J. Biol. Chem..

[77] Hsu D.J., Gao J., Yamaguchi N., Pinzaru A., Wu Q., Mandayam N., Liberti M., Heissel S., Alwaseem H., Tavazoie S. (2023). Arginine Limitation Drives a Directed Codon-Dependent DNA Sequence Evolution Response in Colorectal Cancer Cells. Sci. Adv..

[78] Jansen R.M., Maghe C., Tapia K., Wu S., Yang S., Ren X., Zoncu R., Hurley J.H. (2025). Structural Basis for mTORC1 Regulation by the CASTOR1–GATOR2 Complex. Nat. Struct. Mol. Biol..

[79] Jones R.G., Pearce E.J. (2017). MenTORing Immunity: mTOR Signaling in the Development and Function of Tissue-Resident Immune Cells. Immunity.

[80] Cibrian D., de la Fuente H., Sánchez-Madrid F. (2020). Metabolic Pathways That Control Skin Homeostasis and Inflammation. Trends Mol. Med..

[81] Sarandi E., Krueger-Krasagakis S., Tsoukalas D., Sidiropoulou P., Evangelou G., Sifaki M., Rudofsky G., Drakoulis N., Tsatsakis A. (2023). Psoriasis Immunometabolism: Progress on Metabolic Biomarkers and Targeted Therapy. Front. Mol. Biosci..

[82] Chen T., Liu J., Li S., Wang P., Shang G. (2024). The Role of Protein Arginine N-Methyltransferases in Inflammation. Semin. Cell Dev. Biol..

